# Volume phase transition kinetics of smart *N-n*-propylacrylamide microgels studied by time-resolved pressure jump small angle neutron scattering

**DOI:** 10.1038/s41598-018-31976-4

**Published:** 2018-09-13

**Authors:** Oliver Wrede, Yvonne Reimann, Stefan Lülsdorf, Daniel Emmrich, Kristina Schneider, Andreas Josef Schmid, Diana Zauser, Yvonne Hannappel, André Beyer, Ralf Schweins, Armin Gölzhäuser, Thomas Hellweg, Thomas Sottmann

**Affiliations:** 1 Physical and Biophysical Chemistry, Bielefeld University, Bielefeld, Germany; 2 Institute of Physical Chemistry, University of Cologne, Cologne, Germany; 3 Institute of Physical Chemistry, University of Stuttgart, Stuttgart, Germany; 4 Physics of Supermolecular Systems and Surfaces, Bielefeld University, Bielefeld, Germany; 5 DS/LSS, Institut Laue-Langevin, Grenoble, France

## Abstract

The use of smart colloidal microgels for advanced applications critically depends on their response kinetics. We use pressure jump small angle neutron scattering with supreme time resolution to study the rapid volume phase transition kinetics of such microgels. Utilizing the pressure induced microphase separation inside the microgels we were able to resolve their collapse and swelling kinetics. While the collapse occurs on a time scale of 10 ms, the particle swelling turned out to be much faster. Photon correlation spectroscopy and static small angle neutron scattering unambiguously show, that the much slower collapse can be associated with the complex particle architecture exhibiting a loosely-crosslinked outer region and a denser inner core region. These insights into the kinetics of stimuli-responsive materials are of high relevance for their applications as nano-actuators, sensors or drug carriers. Moreover, the used refined pressure jump small angle neutron scattering technique is of broad interest for soft matter studies.

## Introduction

Smart colloidally stable microgels are promising candidates for various applications, like tunable catalytic environments^1,2^, drug delivery^3^, smart surface coatings^4^, nanoreactors^5^, sensors^6,7^, or nano-actuators^8^. They undergo a reversible volume phase transition (VPT) upon changes of external stimuli, like temperature^9,10^, pH^11–15^, electrochemical potentials^16^, or ionic strength^17,18^. These colloids can be synthesized in a size range from 50 nm up to 1 *μ*m. A comprehensive overview of their properties is given in a recent review by Plamper and Richtering^19^. The structure of these systems was already investigated with scattering^20–25^ and super-resolution techniques^26,27^. The best studied systems in this context are microgels based on *N*-isopropylacrylamide (NIPAM) with a volume phase transition temperature (VPTT) of about 33 °C in water. However, microgel particles based on the isomer *N*-*n*-propylacrylamide (NNPAM)^28,29^, with a VPTT of 22 °C in water, are similar in size and swelling capacity, but are characterized by a very sharp VPT^29^. These particles might even be the first microgels which exhibit a critical discontinuous VPT. For future applications of smart microgels the response kinetics are a crucial issue. The kinetics of the phase transition was up to now investigated only in a non-absolute way^30^, for related macroscopic systems^31–34^, for sub-domains of the particles^35^, or for cononsolvency induced phase transitions^36^. In this work, we utilize the shift of the VPTT by an applied pressure which is observed for microgels^37^ and polymer brushes^38^ to study the swelling/collapse kinetics of NNPAM microgels combining periodic pressure jumps with time-resolved small angle neutron scattering (TR-SANS) experiments exhibiting an up to now unprecedented time resolution. Compared to other techniques to study transition kinetics, the pressure jump technique is the superior method. While e.g. temperature jumps suffer from temperature gradients developing in the sample, the pressure jump technique has the distinct advantage of a rapid and homogeneous adjustment of the new pressure, avoiding the development of gradients. The used high pressure SANS cell, suited for pressures up to 300 bar and temperatures between 5 and 70 °C, is capable of providing periodic bidirectional pressure jumps at a time resolution of 1–2 ms. According to the prediction by Tanaka and Fillmore the swelling time is proportional to the square of the gel dimension^33^. If this relation, which was obtained for spherical macrogels, also holds for microgels, we expect to find relaxation times of the order of milliseconds, which should be resolvable with the above mentioned stroboscopic high pressure SANS cell.

## Results

The poly(NNPAM) microgels with a cross-linker (*N*,*N*-methylenbisacrylamide) content of 4.11 mol% were prepared via an established precipitation polymerization^39^. More details about the synthesis can be found in the materials and methods section below.

### Particle characterization by PCS, HIM and SANS

**Figure 1:**
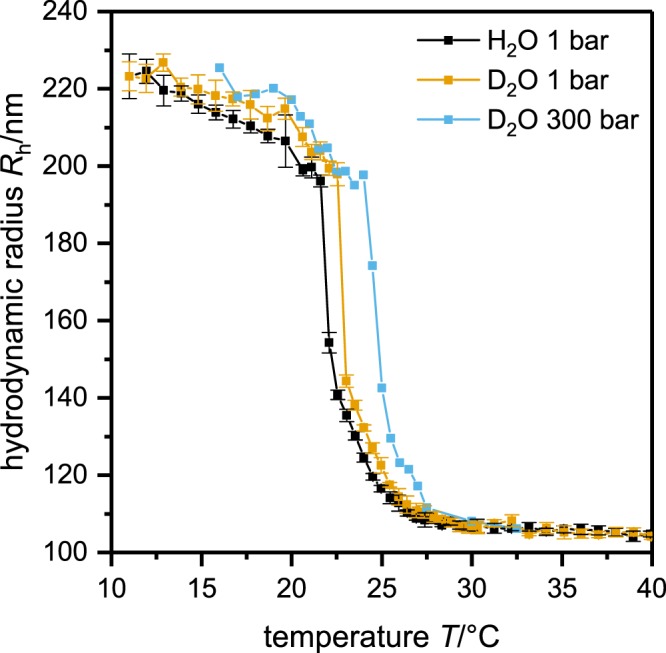
Hydrodynamic radius *R*_h_ as a function of temperature *T* for poly(NNPAM) microgel solutions at ambient pressure in H_2_O and D_2_O and at 300 bar in D_2_O. At temperatures below the VPTT the particles are swollen and above they are in the collapsed state. The transition can be divided into a first sharp collapse and a second broader one. An explanation for this behavior might be the structure of the microgels: The loosely crosslinked dangling ends near the particle surface are collapsing more homogeneously and earlier than the particle core with its internal density distribution. Moreover, the VPTT shifts slightly to higher temperatures (~0.5 K) by changing the solvent from H_2_O to D_2_O. An increase in pressure shifts the VPTT to higher temperatures (~2 K for 300 bar).

Subsequently, the particles were characterized measuring the temperature dependence of the hydrodynamic radius using photon correlation spectroscopy (PCS, for technical details see Materials and Methods section)^40^. For the temperature dependent PCS experiments the particles were prepared in H_2_O and in D_2_O and the experiments were done at ambient pressure and at 300 bar. The resulting hydrodynamic radii are plotted in Fig. 1 as a function of temperature.

**Figure 2:**
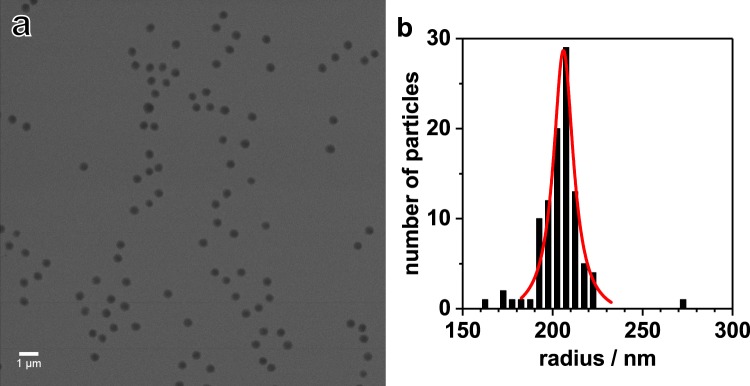
HIM secondary electron image of NNPAM based microgels (**a**) with the radii distribution (red line) (**b**), obtained by analyzing the HIM image. Note that the HIM measurements are performed in high vacuum. Therefore the microgel particles are in the collapsed state.

It is obvious that increasing the pressure from 1 bar to 300 bar, the VPTT of the particles is shifted to higher temperature by approximately 2 K. Moreover, also the exchange of H_2_O by D_2_O leads to a small shift of the VPTT to higher temperatures. The latter effect is similar compared to PNIPAM microgels^41^. Furthermore, the particles were imaged by helium ion microscopy (HIM)^42^. In Fig. 2 a typical HIM image of poly (NNPAM) microgels with the corresponding size distribution is shown. Be aware that the HIM measurements are performed in high vacuum. Therefore the microgel particles are collapsed. Microgel particles deposited on silicon wafers do not retain their spherical shape on the surface but adsorb in a flat form and spread out. Hence the total radius of the particles is comparable to the size of swollen microgels in solution^29,43^. The particle size distribution is rather narrow and the mean radius is 206 ± 7 nm (deviation from FWHM).

**Figure 3:**
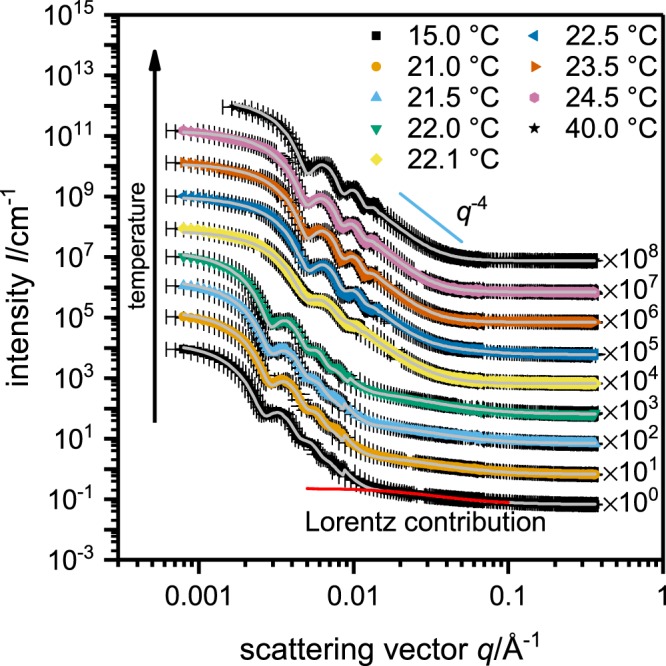
Static SANS data for poly(NNPAM) microgels at ambient pressure and various temperatures. The scattering curves recorded below the VPT can be described by the so called “fuzzy sphere” model with a contribution from the internal network at high *q*. Between 22.0 and 22.1 °C a large shift of the oscillations to higher *q*-values is observed indicating the collapse of the particles. At 22.1 and 22.5 °C the dynamic contribution can be attributed to a collapsed chain (*q*^−3^ dependency instead of *q*^−2^ for a Gaussian chain at lower temperatures). Above 22.5 °C this contribution disappears and the SANS profiles can be fitted with a model for polydisperse homogenous spheres.

The SANS experiments were performed on the D11 spectrometer of the Institut Laue-Langevin (ILL) in Grenoble. Figure 3 shows selected SANS curves at various temperatures. All scattering curves exhibit the typical oscillations obtained in case of monodisperse spherical scatterers. Increasing the temperature, the overall shape of the particles does not change until reaching the VPTT. At temperatures below the VPTT the scattering curves can be described as a fuzzy sphere, with gradually decaying polymer density from the particle center to the surface of the particle^44^, together with a contribution for network fluctuations at higher *q*-values. Between 22.0 and 22.1 °C a large shift of the oscillations to higher *q*-values is observed indicating the collapse of the particles. Furthermore, for 22.1 °C and 22.5 °C the characteristics of the network fluctuation change from those of a Gaussian chain (*q*^−2^ dependency) to a collapsed chain (*q*^−3^ dependency)^45^. In the collapsed state the data are nicely described by a form factor for polydisperse homogenous spheres.

**Figure 4:**
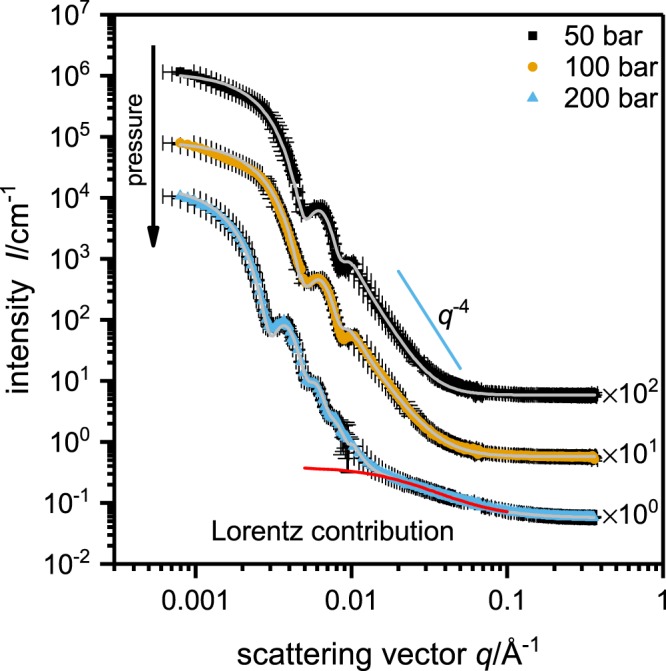
Static SANS data for poly(NNPAM) microgels at a temperature of *T* = 22.8 °C and different pressures. The scattering curves clearly show that the particles are in the shrunken state at 50 and 100 bar, while an increase to 200 bar induces the complete swelling of the particles. The data can be described in the same way as in the case of the temperature dependent measurements.

Figure 4 shows SANS curves obtained at three different pressures and at a temperature of *T* = 22.8 °C. At 50 and 100 bar the microgel particles are in the collapsed state. At 200 bar the particles are fully swollen, this is due to pressure induced cleavage of intramolecular polymer-polymer hydrogen bonds as shown for poly(NIPAM) microgels^37^. The data can be described in the same way as in the temperature dependent measurements. The SANS results are summarized in Table 1. In a previous work by Grobelny *et al*.^37^ even higher static pressures were realized in a small angle X-ray scattering setup. They observed a decrease in size when the pressure is applied to swollen microgels mostly due to a reduction in the fuzzy surface region. When applying high pressure to microgels above the VPTT an increase in particle size indicates a reswelling of the polymer network. From a combination with pressure dependent Fourier transform infrared (FTIR) measurements they concluded that temperature and pressure act antagonistically concerning the VPT. Increasing the pressure leads to better hydration and hence to swelling while increasing the temperature has the inverse effect. At temperatures below the VPTT increasing the pressure leads to a compression of the particles. A pressure induced increase in microgel aggregation, as found by Grobelny *et al*.^37^, could not be observed in the pressure regime used in this experiment. However, the fact that the VPT can be induced by a pressure increase of only 100 bar allows to study the swelling kinetics using pressure jumps.

### Kinetic measurements via time-resolved small angle neutron scattering

**Figure 5:**
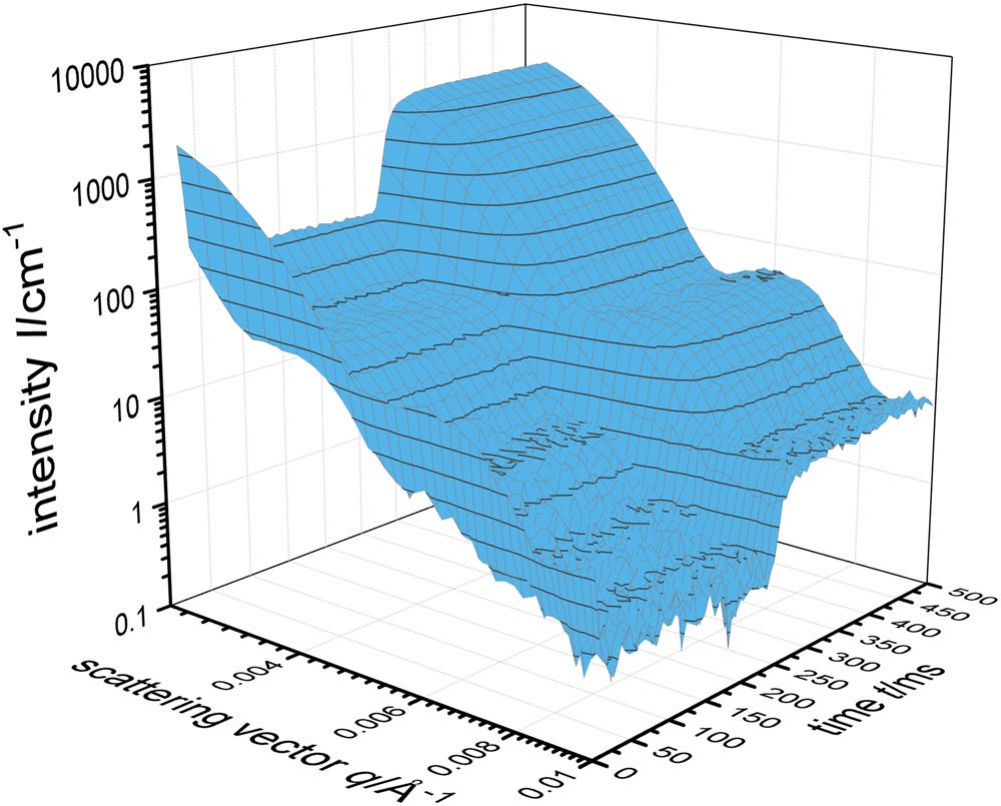
Time resolved small angle neutron scattering data of the pressure jump experiments done between 40 and 200 bar with a resolution of 5 ms for poly(NNPAM) microgels at 22.8 °C.

**Figure 6:**
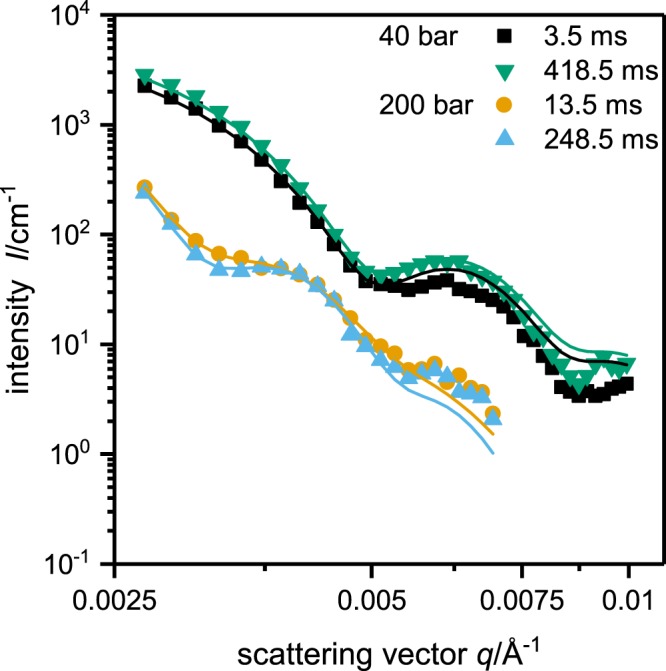
Four typical small angle neutron scattering curves obtained from the pressure jump experiments done between 40 and 200 bar for poly(NNPAM) microgels at 22.8 °C. The scattering curves recorded at 3.5 ms and 418.5 ms (*p* = 40 bar, collapsed state) as well as 13.5 ms and 248.5 ms (*p* = 200 bar, swollen state) were analyzed using the polydisperse homogeneous sphere (black squares and green downwards triangles) or the fuzzy sphere form factor model (orange cycles and light blue upwards triangles).

Hence, subsequently we used a home built stroboscopic high pressure SANS cell and studied the kinetics of pressure induced structural changes performing SANS measurements at a sample to detector distance of 39 m^46^. Repeating the pressure jump cycles (200 bar → 40 bar → 200 bar) 5400 times enabled us to obtain scattering curves with a reasonable statistic (Fig. 5). The pressure induced swelling of the poly(NNPAM) microgels can strikingly be deduced from the shift of the oscillations to smaller *q*-values. Note, that the swelling of the microgels leads to a decreasing scattering contrast and a shift of the Guinier region, which entails the lower scattering intensity. Figure 6 shows four typical SANS curves recorded at 3.5 ms and 418.5 ms (*p* = 40 bar) as well as 13.5 ms and 248.5 ms (*p* = 200 bar). The fact, that the scattering curves recorded at the same pressure but at different times match each other almost quantitatively, illustrates the quality of the data. Furthermore, it can be seen, that the two form factor models, i.e. the polydisperse homogeneous sphere and the fuzzy sphere model, describe the scattering data almost quantitatively. Only small deviations between the data recorded at high pressure in the swollen state and the fuzzy sphere model become obvious at large *q*-values.

**Figure 7:**
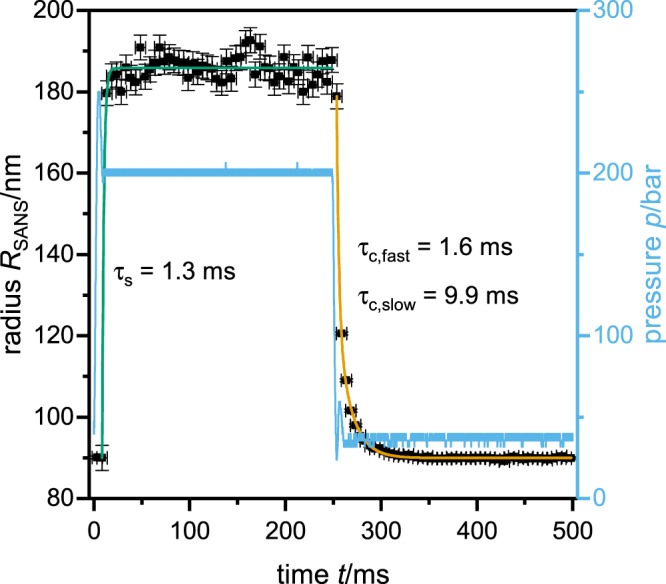
Particle radius *R*_SANS_ and respective pressure *p* against cycle time *t* for the pressure jump experiments done between 40 and 200 bar at 22.8 °C. For the particle swelling a sharp transition is found. When described with an exponential function (green line) a time constant of *τ*_*s*_ = 1.3 ms is obtained, which is the resolution limit of the experiment. Thus, the real time constant of the swelling could be even faster. The deswelling is much slower and can be described with a biexponential decay (orange line) with time constants of *τ*_c,fast_ = 1.6 ms and *τ*_c,slow_ = 9.9 ms. The experimental time is corrected for the neutron flight time (59 ms, corresponding to neutrons with 6 Å wavelength) and the delay in the pressure jump (24 ms).

Having analyzed each SANS curve individually, the obtained particle radius is plotted against the cycle time together with the actual pressure (Fig. 7). The particle radius is of the order of 185 nm in the swollen state at 200 bar, decreasing to 90 nm after the pressure jump to 40 bar. As can be seen, the particle swelling (around 0 ms on the time axis in Fig. 7) is much faster than the particle collapse (starting at 250 ms). A time constant of *τ*_*s*_ = 1.3 ms is obtained from a fit of the swelling kinetics with a monoexponential function. However, as this this time constant is the resolution limit of the experiment, the real time constant of the swelling could be even faster. Besides this flaw the result is in good agreement with the predictions based on the theory by Tanaka and Fillmore^33^. This theory predicts swelling times for gels of this size to be *τ* = 1.3 ms. Interestingly, The microgel deswelling observed at a cycle time of 250 ms can only be quantitatively described by a biexponential function with a time constant of 1.6 ms for the fast and a time constant of 9.9 ms for the slow deswelling process. The fast contribution in the deswelling curve is on the same time scale as the microgel swelling. When compared with other experiments the deswelling time is in very good agreement with the fast deswelling time constant observed for the solvent-exchange induced transitions by Keidel *et al*. These authors have observed a biexponential particle collapse as well, but their second time constant is more than one order of magnitude larger (227 ms). Which could be caused by the difference in the experimental technique, as the solvent exchange itself might be slower than the pressure propagation^36^. Wang *et al*. observed much faster (0.39 *μ*s) deswelling kinetics with a laser induced temperature jump. They explained these very fast kinetics with an incomplete collapse of the microgel particles as the transmittance during the jump changes only by 3–4% of the change of a full collapse^35^. Suaréz *et al*. found swelling kinetics in the range of 30 to 600 ms for minigels (8–60 *μ*m in diameter). In comparison to our results and those by Tanaka *et al*. these kinetics are faster than expected. This might be caused by the different synthetic conditions using an inverse emulsion polymerization. From the observed time constants the network diffusion coefficients *D* could be calculated by the following equation^33^:

1 $$D=\frac{{r}_{{\rm{final}}}}{\tau }$$

For the swelling a diffusion coefficient of 2.6·10^−11^ m^2^/s can be given as lower limit, while for the two deswelling time constants, diffusion coefficients of 5.1·10^−12^ m^2^/s and 8.1·10^−11^ m^2^/s are obtained. The values for the swelling and the faster deswelling process are of the same order of magnitude as the collective network diffusion coefficient for microgels obtained by Hellweg *et al*. through neutron spin echo spectroscopy (3.16·10^−11^ m^2^/s for a microgel with 5 mol% crosslinker)^47^.

## Discussion

Thus, these experiments unambiguously show that the particle collapse/deswelling is much slower (by a factor of at least 10) than the swelling process. The origin of this behavior is most-likely related to an inhomogeneous collapse of the microgels due to the non-equal distribution of the cross-linker content throughout the whole particle. A similar behavior was already observed for the temperature induced deswelling of macroscopic gels^34^ and the solvent-exchange induced transition in microgels^36^. In both cases the formation of an impermeable layer at the water-gel interface leads to a significantly retarded deswelling. In combination with the PCS and the static SANS results only the picture of an early collapsing outer region (sharp transition in PCS; *q*^−3^-dependency in SANS; slower deswelling kinetics) and a denser inner core region with a higher cross-linker content makes sense, as this higher cross-linked region should contain shorter chains between the cross-links and therefore a higher VPTT for each chain^48^. Hence, a fast collapse of the loosely cross-linked outer part of the microgels might lead to a reduction of the deswelling speed due to a decrease of the permeability of the already collapsed outer parts of the microgel particles. This would explain both the presence of a non-mono exponential deswelling and the time constant difference between the swelling and the deswelling of the microgel particles. A two-step collapse was also observed by Keidel *et al*. for cononsolvency induced phase transitions^36^. Their experiments show a first-step on a similar timescale, but a much slower second step, which might be caused by the diffusion limited collapse inducement. The difference in the transition kinetics is in contrast to the latest simulation data by Nikolov *et al*.^49^, where the opposite behavior was found. However, the very fast deswelling kinetics in their case are due to highly porous intermediate states which accelerate the transition. If these states are not accessible due to a rather high content and homogeneous distribution of the crosslinker the collapse of the particle is expected to be slower. In conclusion in this study we present the determination of the swelling and deswelling kinetics of sub-micrometer acrylamide based microgels. Utilizing the periodic pressure jump technique, the swelling and collapse kinetics of microgels were determined by time-resolved SANS. Therewith time constants for the deswelling (1.6 and 9.9 ms) could be determined. Due to the resolution limit of the experiment, the time constant of *τ*_*s*_ ≤ 1.3 ms found for the swelling has to be treated as an upper limit. These very short times clearly show the potential of poly(NNPAM) microgels for applications as sensors and nano-actuators.

## Materials and Methods

### *N*-*n*-propylacrylamide synthesis

*N*-*n*-propylacrylamide (NNPAM) was synthesized by a Schotten-Baumann reaction following the protocol described by Hirano *et al*.^50^. A solution of 0.5 mol of acryloyl chloride (Alfa Aesar, Ward Hill, U.S.A) in 200 ml dichloromethane (HiPerSolv, VWR, Center Valley, U.S.A.) was added dropwise over 3 hours to a mechanically stirred and ice-bath cooled solution of 0.6 mol of *n*-propylamine (purum, Sigma-Aldrich, St-Louis, U.S.A.) and 0.5 mol of triethylamine (for synthesis, Carl Roth GmbH & Co. KG, Karlsruhe, Germany) in 200 ml dichloromethane. The mixture was kept stirring at room temperature for 24 hours and filtrated with a Büchner funnel afterwards. The filtrate was washed with 10% sodium hydrogen carbonate and dried over sodium sulfate. The solvent was removed by rotary evaporation and the final product obtained after vacuum distillation (115 °C, 10 mbar) with a yield of 30–40%.

### Microgel synthesis

The *N*-*n*-propylacrylamide based microgels were prepared via an established precipitation polymerization^39^. 11.55 mmol of the monomer were added together with 0.475 mmol of the crosslinker (99%, *N*,*N*’-methylenebisacrylamide, BIS, Sigma-Aldrich, St-Louis, U.S.A.) and 0.0172 mmol of sodium dodecylsulfate (ultra purum, Carl Roth GmbH & Co. KG, Karlsruhe, Germany) to 150 mL purified water (Arium pro VF system, Sartorius AG, Göttingen, Germany). The solution was heated to 70 °C, purged with nitrogen gas and the reaction was initiated by addition of 1 mL of a 0.4 M ammonium persulfate (ACS reagent, Sigma-Aldrich, St-Louis, U.S.A.) solution. The resulting polymer particles were purified by 5 cycles of centrifugation, decantation and redispersion in purified water. This procedure removes the surfactant and low molecular weight impurities from the microgel suspension.

### Small angle neutron scattering experiments

The SANS measurements were carried out at the D11 spectrometer at the Institut Laue-Langevin (ILL) in Grenoble, France. The wavelength was adjusted to 6 and 13 Å with a wavelength spread of $\delta \lambda /\lambda =0.09$ (full width at half maximum; FWHM), specified by the ILL for the D11 instrument. Static measurements were performed at sample-to-detector distances of 1.75, 10 and 39 m using collimation lengths of 10.5, 10.5 and 40.5 m, respectively. The transmission was determined at 8 m sample-to-detector distance with a collimation of 10.5 m. While the temperature dependent measurements where performed in a home built cell-holder (Hellma-quartz 404 glass cells, path length of 2 mm, Hellma Analytics, Müllheim, Germany) which exhibits a very high temperature stability (${\rm{\Delta }}T=\pm \,0.02$ K). The experiments at elevated pressure were performed in a home built stroboscopic high pressure SANS cell (SHP-SANS, see below). Note, that the pressure jump experiments were performed at a sample-to-detector distance of 39 m and a collimation length of 40.5 m. For all experiments a circular beam-defining aperture of 13 mm diameter was used in front of the sample holder. All measurements were done at a concentration of 0.5 wt% in D_2_O. The solvent was exchanged by several centrifugation-redispersion cycles similar to the purification after the particle synthesis. The scattering experiments were performed between 15 and 40 °C changing the temperature manually. Before each experiment the setup/sample equilibrated at the selected temperature for at least 30 minutes. The raw data were normalized to absolute scale using the incoherent scattering of H_2_O as secondary calibration standard. The scattering intensity ${I}_{{{\rm{H}}}_{2}{\rm{O}}}$ of H_2_O was determined at sample-to-detector distances of 1.75 and 10 m. The overlap between data collected at 10 and 39 m was used for the normalization of the data obtained at a sample-to-detector distance of 39 m for which the calibration measurement of the H_2_O-scattering with adequate signal-to-noise ratio would have been too time-consuming. Furthermore, the scattering intensities of the empty cells, *I*_EC_ and ${I}_{{\rm{EC}},{{\rm{H}}}_{{\rm{2}}}{\rm{O}}}$, and the instrumental background *I*_BG_ were measured. In addition the respective transmissions *T*_*i*_ and the neutron path ways of H_2_O ${d}_{{{\rm{H}}}_{2}{\rm{O}}}$ and sample *d*_SA_ were taken into account in order to determine the differential scattering cross section per unit volume of the sample, according to eq. 2.

2 $${(\frac{d\Sigma }{d{\rm{\Omega }}})}_{{\rm{SA}}}(q)=\frac{{I}_{{\rm{SA}}}-{I}_{{\rm{BG}}}-\frac{{T}_{{\rm{SA}}}}{{T}_{{\rm{EC}}}}\cdot ({I}_{{\rm{EC}}}-{I}_{{\rm{BG}}})}{{I}_{{{\rm{H}}}_{2}{\rm{O}}}-{I}_{{\rm{BG}}}-\frac{{T}_{{{\rm{H}}}_{2}{\rm{O}}}}{{T}_{{\rm{EC}},{{\rm{H}}}_{2}{\rm{O}}}}\cdot ({I}_{{\rm{EC}},{{\rm{H}}}_{2}{\rm{O}}}-{I}_{{\rm{BG}}})}\cdot \frac{{T}_{{{\rm{H}}}_{2}{\rm{O}}}{d}_{{{\rm{H}}}_{2}{\rm{O}}}}{{T}_{{\rm{SA}}}{d}_{{\rm{SA}}}}\cdot {(\frac{d\Sigma }{d{\rm{\Omega }}})}_{{{\rm{H}}}_{2}{\rm{O}}}$$

Data normalization to absolute scale was performed by using the software LAMP® at the ILL, taking into account also the dead time of the detector. One-dimensional scattering curves were obtained from the masked and radially averaged two-dimensional detector image.

### Stroboscopic high pressure SANS cell

The pressure jump measurements were performed in a home built stroboscopic high pressure SANS cell (SHP-SANS)^46^. The cell, which can be filled with liquids as well as gases, was designed by the mechanical workshop of the Institute of Physical Chemistry at the University of Cologne.

**Figure 8:**
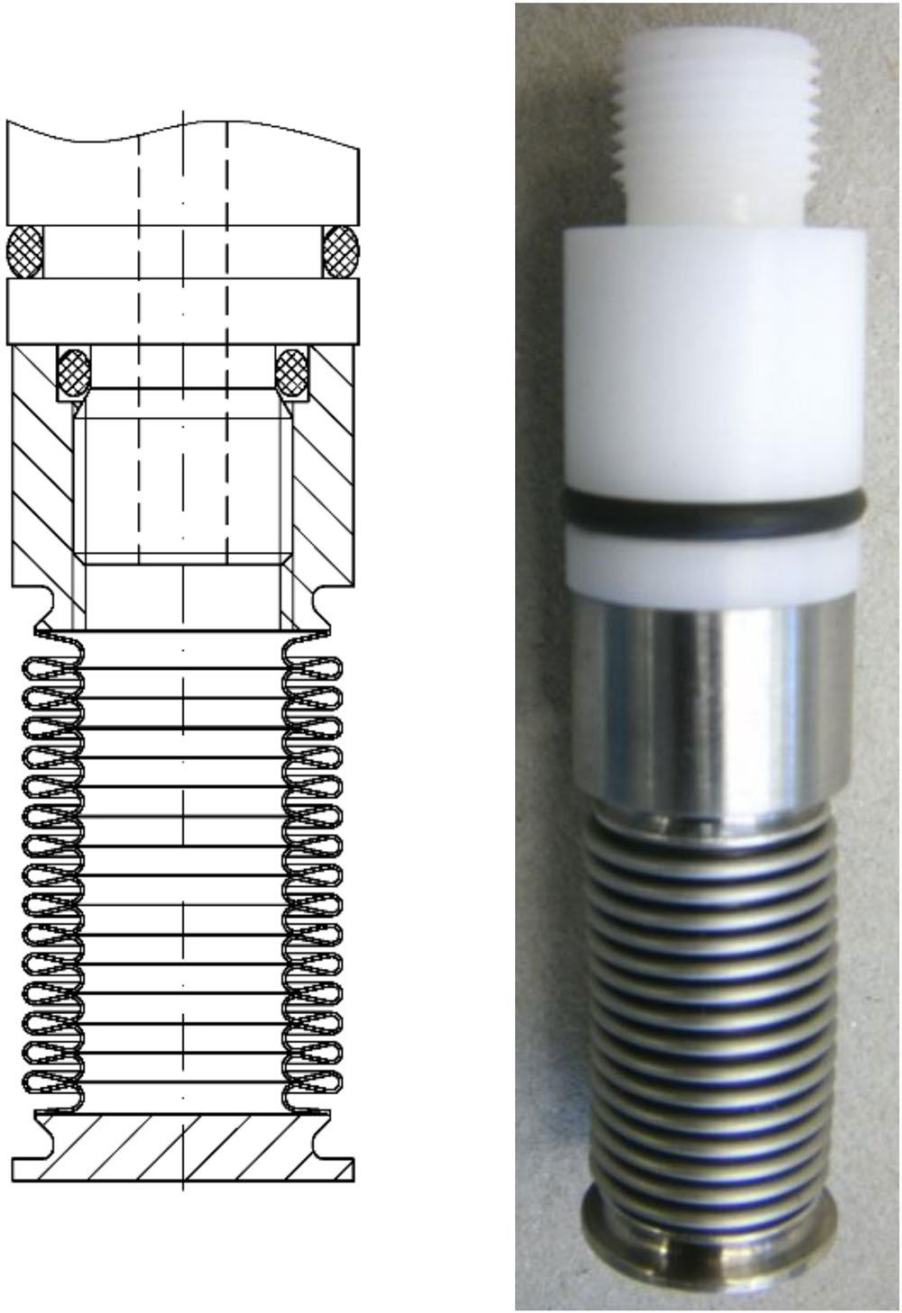
Technical drawing (left) and photograph (right) of the hollow metal bellow^57,58^. The bellow is attached to an also hollow tuneable piston. A sealing ring (NBR 90) closes the cell volume from the top. Flowing hydraulic oil, which is controlled by a hydraulic pump, through the piston into the bellow its volume is adjustable. Since the bellow is located inside the cell, periodic elongation and shrinkage of the bellow leads to periodic changes of the cell volume and therewith the pressure. The neutron path way length is not influenced by the change in cell volume. Reprinted with permission from (Pütz, Y. *CO*_2_*-microemulsions with additives: Phase behaviour, microstructure and pressure-induced kinetics* (Cuvillier Verlag, Göttingen, 2015)). Copyright (2015) Cuvillier Verlag.

It generates periodic pressure cycles with adjustable amplitude and frequency for periods up to several hours. The core of the cell, made of bronze, is equipped with two parallel sapphire windows (thickness 12 mm; neutron path way is 2 mm and not influenced by the pressure changes) allowing to check the phase behavior before and after the SANS-measurement, while an adequate transmission of neutrons is provided at the same time. The entrance and exit window have a diameter of ${d}_{{\rm{entrance}}}=18$ mm and ${d}_{{\rm{exit}}}=32$ mm, respectively. Above and below the windows the sample volume (which overall amounts to about 13 cm^3^) becomes cylindrical (*d* = 16 mm). A pressure probe is inserted into the cell allowing for measuring the pressure with an accuracy of ${\rm{\Delta }}p=\pm \,5$ bar. A tunable hollow piston connected to an also hollow metal bellow (Fig. 8) closes the cell volume from the top (via a sealing ring (NBR 90)) and allows adjusting the sample volume and thus the pressure. The length of the bellow is controlled by a connected hydraulic pump having two outlets, through which hydraulic oil is pumped each with an adjustable pressure (*p*_1_, *p*_2_ < 500 bar).

**Figure 9:**
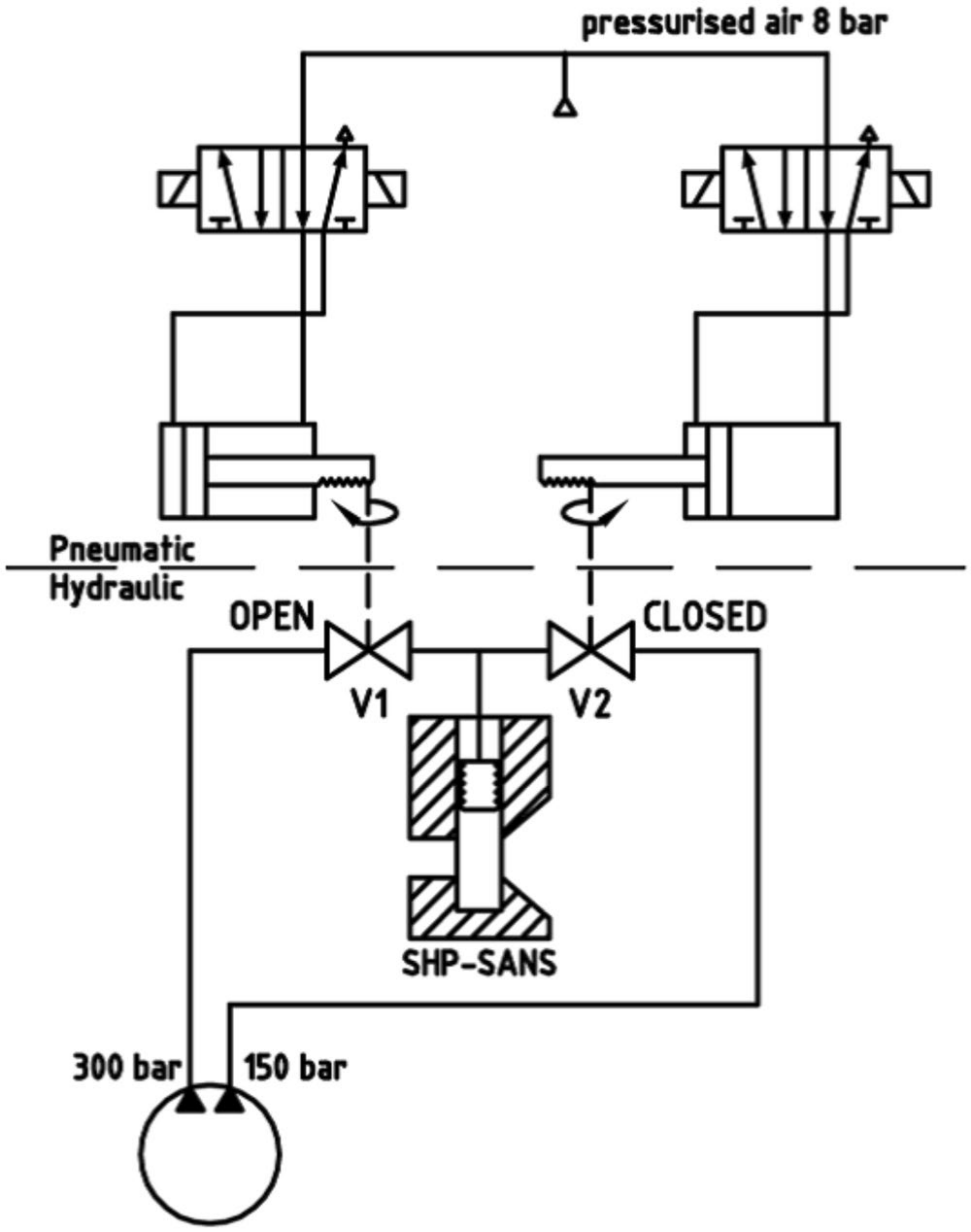
Pneumatic and hydraulic circuit diagram, showing how the pressure inside the cell is controlled^58^. The hydraulic pump (circle) has two outlets, through which hydraulic oil is pumped each with an adjustable pressure (*p*_1_, *p*_2_ < 500 bar). The valves V1 and V2 control, which of the two flows is connected to the piston/bellow and thus the pressure within the cell. V1 and V2 are operated by an antagonistic circuit of four pneumatic valves, one pneumatic valve each to open or close one hydraulic valve, enabling the generation of pressure cycles with adjustable frequency of $\nu  < 23$ Hz. Reprinted with permission from (Pütz, Y. *CO*_2_*-microemulsions with additives: Phase behaviour, microstructure and pressure-induced kinetics* (Cuvillier Verlag, Göttingen, 2015)). Copyright (2015) Cuvillier Verlag.

Hydraulic ball valves (V1 and V2, see Fig. 9) control which of the two flows is connected to the piston/bellow. Afterwards the valve is closed to obtain a constant pressure. Via opening and closing of the other valve, a lower or larger flow leads to a shrinkage or extension of the bellow and therewith to a lower or larger pressure in the cell, respectively. Therewith periodic pressure cycles can be easily generated inside the cell. The hydraulic ball valves V1 and V2 are operated by an antagonistic circuit of four pneumatic valves, one pneumatic valve each to open or close one hydraulic valve. These valves are controlled using compressed air (*p* = 8–10 bar). Therewith, switching times of 11 ms could be obtained for the hydraulic ball valves. Since a pressure cycle consists of four switching operations (V1 open, V1 close, V2 open, V2 close), the maximum adjustable frequency amounts to $\nu \approx 23$ Hz. Note, that the pressure cycles can be repeated as often as necessary.

**Figure 10:**
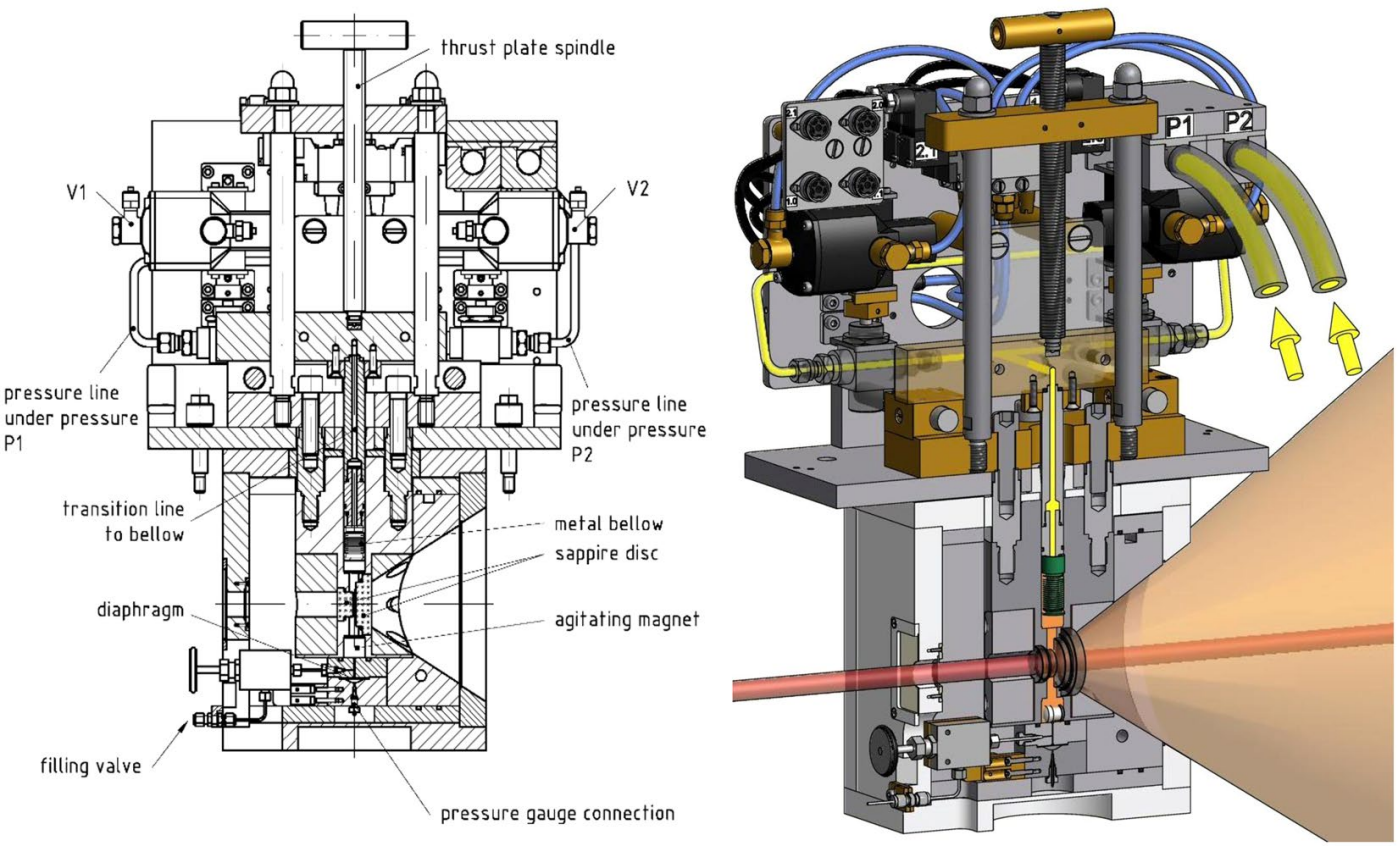
Mechanical cross section (left) and 3D-rendered cross section (right) through the SHP-SANS cell^57,58^. The thrust plate spindle moves the piston, while the metal bellow (shown in green, right) and piston allows the variation of the cell volume (orange) and thus the pressure. The hydraulic oil is shown in yellow. The cell is equipped with two sapphire windows (thickness 12 mm) installed in parallel at a distance of 2 mm. The neutron beam (highlighted in red) enters on the left. The conical shape on the right, allows the scattering up to an angle of $\theta ={64}^{\circ }$. The cell volume $V\approx 13$ cm^3^ is sealed via the piston and a diaphragm at the bottom. Reprinted with permission from (Müller, A. *Preparation of polymer nano-foams: templates, challenges and kinetics* (Cuvillier Verlag, Göttingen, 2013) and Pütz, Y. *CO*_2_*-microemulsions with additives: Phase behaviour, microstructure and pressure-induced kinetics* (Cuvillier Verlag, Göttingen, 2015).). Copyright (2013 and 2015) Cuvillier Verlag.

To avoid overheating, water and two fans cool the hydraulic pump. Looking at the pressure profiles (see Fig. 7) the pressure oscillates slightly around the envisaged pressure for a short period after the pressure jump. Thus, although the envisaged pressure is reached for the first time after about 3 ms, it becomes constant after 10 ms. Note, that the pressure profile can be finely adjusted by the hydraulic pressures *p*_1_ and *p*_2_ (hydraulic pump) and the switching times of the pneumatic valves, which are operated by a LABVIEW-based software program displaying also the values of *p*_1_ and *p*_2_. Beside it transmits a trigger signal to the D11 instrument. Thus, having defined and started a kinetic count, the detector starts counting upon the trigger signal. Two sketches of the SHP-SANS cell, a mechanical cross section (left) and a 3D-rendered cross section (right) are displayed in Fig. 10. Looking at the 3D-rendered cross section, the neutron beam (highlighted in red) is found to enter the cell on the left hand side via the small sapphire window. On the right side, a larger sapphire window and the conical shape of the SHP-SANS cell exhibiting an opening angle of $\theta ={64}^{\circ }$, allow a full illumination of the detector even at shortest sample-to-detector distances. Note, that the sample volume is marked orange. The hydraulic circuit which connects metal bellow and piston with the hydraulic pump via the hydraulic valves V1 and V2 is marked yellow. At the bottom the cell is sealed by a valve and a diaphragm. An agitating magnet inside of the cell volume enables homogenization of the sample. The diaphragm separates the cell volume from a miniature pressure transducer (Type 81530-500, Burster, Germany) via an air-free water reservoir, allowing to determine the pressure with an accuracy of ${\rm{\Delta }}p=\pm \,5$ bar. The pressure is displayed and recorded by a PC Oscilloscope PicoScope 3204. The temperature is measured via a temperature sensor (PT100 A class, RS Components GmbH, Germany) close to the cell volume. The core of the cell is placed in a thermo-casing, made of aluminium and isolated by polyoxymethylene. By connecting the thermo-casing to a thermostat the temperature of the SHP SANS cell can be varied between 5 and 70 °C with an accuracy of ${\rm{\Delta }}T=\pm \,0.1$ °C. The tunable combination of piston and metal bellow allows varying the pressure between 1 and 300 bar.

### Pressure jump experiments

In the kinetic experiment described in the paper, 5400 pressure cycles were performed using the SHP-SANS cell. Each cycle, consisting of a jump from low to high pressure and a jump back from high to low pressure, lasted 600 ms. *p*_high_ and *p*_low_ were adjusted to 200 bar and 50 bar, respectively. *p*_high_ was kept for 250 ms, while *p*_low_ was kept for 350 ms. Thereby, at *p*_low_ the detector signal was only recorded for 250 ms. The additional 100 ms were used to ensure the data transfer. The whole detection cycle was divided into 100 detector slices with a temporal width of 5 ms each. For the data processing the standard D11-software LAMP was used. The experimental time was corrected for the delay in the pressure jump and the neutron flight time ${t}_{{\rm{flight}}}$ calculated from the neutron velocity ${v}_{{\rm{neutron}}}$ and the sample-to-detector distance *d*_SD_.

3 $${v}_{{\rm{n}}{\rm{e}}{\rm{u}}{\rm{t}}{\rm{r}}{\rm{o}}{\rm{n}}}=\frac{h}{m\cdot \lambda }=\frac{6.63\cdot {10}^{-34}\frac{{\rm{k}}{\rm{g}}\cdot {{\rm{m}}}^{2}}{{\rm{s}}}}{1.67\cdot {10}^{-27}\,{\rm{k}}{\rm{g}}\cdot 6\,{\rm{\AA }}}=659.34\frac{{\rm{m}}}{{\rm{s}}}$$

4 $${t}_{{\rm{f}}{\rm{l}}{\rm{i}}{\rm{g}}{\rm{h}}{\rm{t}}}=\frac{{d}_{{\rm{S}}{\rm{D}}}}{{v}_{{\rm{n}}{\rm{e}}{\rm{u}}{\rm{t}}{\rm{r}}{\rm{o}}{\rm{n}}}}=\frac{39.09\,{\rm{m}}}{659.34\frac{{\rm{m}}}{{\rm{s}}}}=59.29\,{\rm{m}}{\rm{s}}$$

The time resolution of the experiment Δτ is limited by the wavelength distribution (${\rm{\Delta }}\lambda /\lambda =0.09$, FWHM). The different velocities of neutrons of different wavelength lead to a spreading of an ensemble of neutrons scattered at a given time. The time resolution can be directly calculated from the neutron flight time and the wavelength distribution:

5 $${\rm{\Delta }}t={t}_{{\rm{flight}}}\cdot \frac{{\rm{\Delta }}\lambda }{\lambda }=59.29\,{\rm{ms}}\cdot 0.09=5.34\,{\rm{ms}}$$

The minimal lifetime Δτ_min_ that can be obtained from the data can therefore be estimated by calculating the lifetime from the time resolution and the standard deviation of the obtained radius ${\sigma }_{{R}_{\mathrm{SANS}}}$:

6 $${\tau }_{{\rm{\min }}}=\frac{{\rm{\Delta }}t}{\mathrm{ln}(\frac{1}{{\sigma }_{{R}_{{\rm{SANS}}}}})}=\frac{5.34\,{\rm{ms}}}{\mathrm{ln}(60.74)}=1.30\,{\rm{ms}}$$

### Helium Ion Microscopy (HIM)

For helium ion microscopy (HIM), we deposited the microgel particles on silicon wafers (Siegert Wafer GmbH, Aachen, Germany), dried them and imaged them using a Zeiss Orion Plus helium ion microscope (Carl Zeiss, Oberkochen, Germany). The HIM has the advantage that no conductive coating of the sample is necessary and has a good contrast for organic material^42,51^. The distribution of the radius was analyzed using the program FIJI^52^.

### Photon Correlation Spectroscopy (PCS)

**Figure 11:**
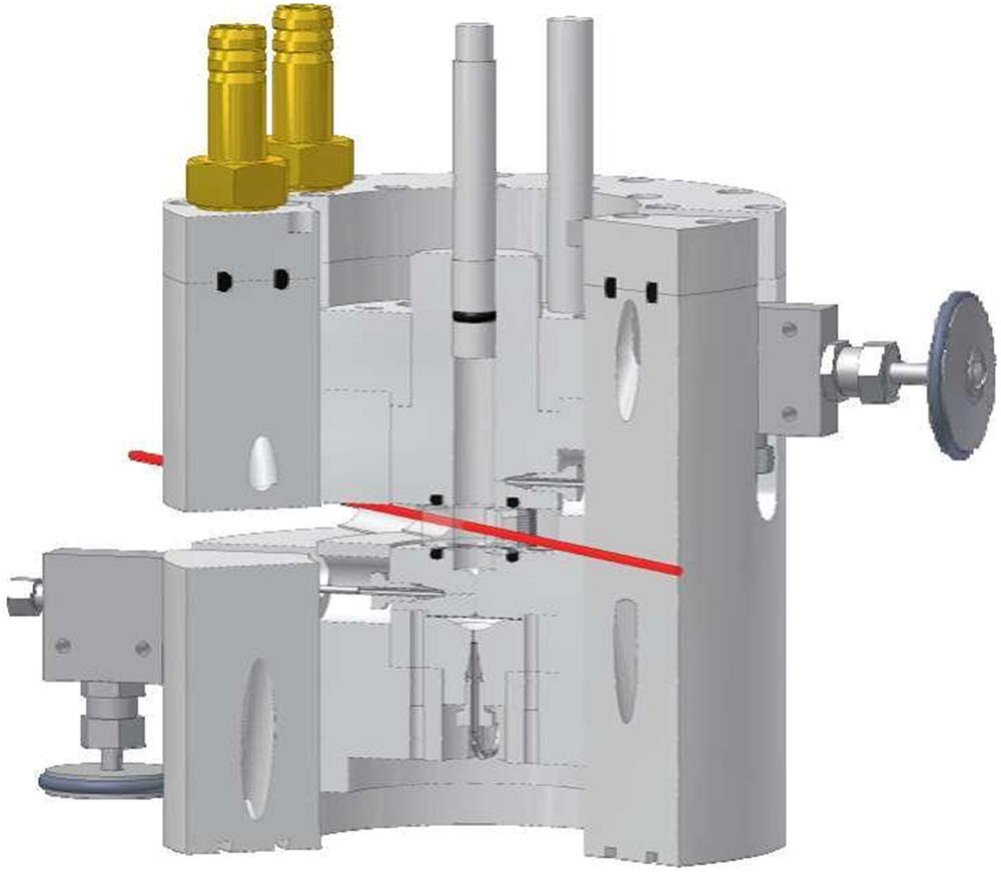
Cross-section through the high pressure photon correlation spectroscopy setup. The sapphire disk in the middle is passed by a laser beam and allows PCS measurements from 30 to 150°. Reprinted with permission from (Pütz, Y. *CO*_2_*-microemulsions with additives: Phase behaviour, microstructure and pressure-induced kinetics* (Cuvillier Verlag, Göttingen, 2015).). Copyright (2015) Cuvillier Verlag.

The photon correlation spectroscopy (PCS) measurements at ambient pressures where performed on a fixed angle setup (60°) in pseudo-crosscorrelation mode with two photomultipliers (ALV/SO-SIPD, ALV-GmbH, Langen, Germany) and an ALV-6010 multiple-tau correlator (ALV-GmbH, Langen, Germany). A Helium-Neon-Laser (632.8 nm, 21 mW, Thorlabs, Newton, USA) was used to illuminate the sample having a concentration below 0.01 wt% to avoid multiple scattering. The temperature inside the decaline matching bath was controlled using a thermostat and equilibrated for 25 minutes for each temperature. The pressure dependent measurements were performed on a modified BI-200SM system (Brookhaven Instruments Corporation, Brookhaven, USA), in which a sapphire disk with an outer diameter of 2.5 cm and an inner diameter of 1 cm is implemented. It allows pressure and angular dependent measurements up to 300 bar and between 30° and 150°, respectively. A cross section of this setup is shown in Fig. 11. Similar to the SHP-SANS cell the volume and pressure is controlled by a tunable piston which closes the cell from the top. A diaphragm separates the cell volume from a miniature pressure transducer (Type 81530-500, Burster, Germany) via an air-free water reservoir, allowing to determine the pressure with an accuracy of ${\rm{\Delta }}p=\pm \,5$ bar. To control the temperature of the sample, the outer ring of the sample holder can be connected to a thermostat bath using the two connections at the top Table 1.

**Table 1:** Parameters obtained from fitting the static SANS scattering curves shown in Fig. 3 and 4.

*T*/°C	*p*	*R*_SANS_/nm	*σ*_surf_/nm	*σ*_poly_/%	*ξ*/nm
15.0	ambient	200	13	8.3	3.9*
21.0	ambient	186	14	8.4	4.4*
21.5	ambient	184	14	9.0	4.6*
22.0	ambient	183	13	6.7	5.5*
22.1	ambient	88	—	13.2	8.5
22.5	ambient	90	—	8.2	1.6
23.5	ambient	91	—	7.0	—
24.5	ambient	92	—	7.6	—
40.0	ambient	91	—	6.2	—
22.8	50 bar	90	—	9.2	—
22.8	100 bar	90	—	9.8	5.9
22.8	200 bar	173	11	7.8	4.6*

For low temperatures and high pressures the scattering curve can be described by the fuzzy-sphere model, which combines a highly cross-linked core with a fuzzy shell and a contribution which accounts for the network motion. For high temperatures and low pressures the model of a homogeneous sphere is sufficient. *R*_SANS_ is the radius of the whole particle and a combination of the fitted radius *R* and the fuzziness *ρ*_surf_: ${R}_{{\rm{S}}{\rm{A}}{\rm{N}}{\rm{S}}}=R+2\cdot {\sigma }_{{\rm{s}}{\rm{u}}{\rm{r}}{\rm{f}}}$. The network motion (correlation length *ξ*) is described by a Lorentzian-type function ${\mathrm{(1}+{(q\cdot \xi )}^{n})}^{-1}$, with *n* = 2 for a Gaussian chain* or *n* = 3 for a collapsed chain.

The PCS measurements were analyzed using Laplace inversion of the experimental intensity time correlation functions by means of the program CONTIN^53,54^ and the cumulants method^55,56^.

## References

[1] Lu Y, Mei Y, Ballauff M, Drechsler M (2006). Thermoresponsive core-shell particles as carrier systems for metallic nanoparticles. J. Phys. Chem. B.

[2] Lu Y, Mei Y, Drechsler M, Ballauff M (2006). Thermosensitive core-shell particles as carriers for Ag nanoparticles: Modulating the catalytic activity by a phase transition in networks. Angew. Chem..

[3] Nayak S, Lyon LA (2005). Soft nanotechnology with soft nanoparticles. Angew. Chem. Int. Ed..

[4] Uhlig K (2016). Patterned thermoresponsive microgel coatings for noninvasive processing of adherent cells. Biomacromolecules.

[5] Das M, Sanson N, Kumacheva E (2008). Zwitterionic poly(betaine-*N*-isopropylacrylamide) microgels: Properties and applications. Chemistry of Materials.

[6] Zhang QM, Wang W, Su Y-Q, Hensen EJM, Serpe MJ (2016). Biological imaging and sensing with multiresponsive microgels. Chemistry of Materials.

[7] Liu T (2009). Enhancing detection sensitivity of responsive microgel-based Cu(ii) chemosensors via thermo-induced volume phase transitions. Chemistry of Materials.

[8] Zeiser M, Freudensprung I, Hellweg T (2012). Linearly thermoresponsive core-shell microgels: Towards a new class of nanoactuators. Polymer.

[9] Pernia Leal M (2012). Controlled release of doxorubicin loaded within magnetic thermo-responsive nanocarriers under magnetic and thermal actuation in a microfluidic channel. ACS Nano.

[10] Clarke KC, Dunham SN, Lyon LA (2015). Core/shell microgels decouple the pH and temperature responsivities of microgel films. Chemistry of Materials.

[11] Serpe MJ, Lyon LA (2004). Optical and acoustic studies of pH-dependent swelling in microgel thin films. Chemistry of Materials.

[12] Suzuki D, Tsuji S, Kawaguchi H (2007). Janus microgels prepared by surfactant-free pickering emulsion-based modification and their self-assembly. Journal of the American Chemical Society.

[13] Kim J, Serpe MJ, Lyon LA (2004). Hydrogel microparticles as dynamically tunable microlenses. Journal of the American Chemical Society.

[14] Dai Y (2012). Up-conversion cell imaging and pH-induced thermally controlled drug release from NaYF4:Yb3+/Rr3+ @hydrogel core-“shell hybrid microspheres. ACS Nano.

[15] Schachschal S (2010). Polyampholyte microgels with anionic core and cationic shell. Macromolecules.

[16] Mergel O, Wünnemann P, Simon U, Böker A, Plamper FA (2015). Microgel size modulation by electrochemical switching. Chemistry of Materials.

[17] Karg M (2008). Temperature, pH, and ionic strength induced changes of the swelling behavior of pNIPAM-poly(allylacetic acid) copolymer microgels. Langmuir.

[18] Fernandez-Nieves A, Fernandez-Barbero A, de las Nieves FJ (2001). Salt effects over the swelling of ionized mesoscopic gels. J. Chem. Phys..

[19] Plamper FA, Richtering W (2017). Functional microgels and microgel systems. Acc. Chem. Res..

[20] Mears SJ, Deng Y, Cosgrove T, Pelton R (1997). Structure of sodium dodecyl sulfate bound to a poly(NIPAM) microgel particle. Langmuir.

[21] Crowther HM (1999). Poly(NIPAM) microgel particle de-swelling: a light scattering and small-angle neutron scattering study. Colloids and Surfaces A: Physicochemical and Engineering Aspects.

[22] Kratz K, Hellweg T, Eimer W (2001). Structural changes in pNIPA microgel particles as seen by SANS, DLS, and EM techniques. Polymer.

[23] Fernandez-Barbero A, Fernandez-Nieves A, Grillo I, Lopez-Cabarcos E (2002). Structural modifications in the swelling of inhomogeneous microgels by light and neutron scattering. Phys. Rev. E.

[24] Saunders BR (2004). On the structure of poly(*N*-isopropylacrylamide) microgel particles. Langmuir.

[25] Fernandez-Nieves A, de las Nieves F, Fernandez-Barbero A (2004). Static light scattering from microgel particles: Model of variable dielectric permittivity. J. Chem. Phys..

[26] Gelissen APH (2016). 3D structures of responsive nanocompartmentalized microgels. Nanoletters.

[27] Conley GM, Nöjd S, Braibanti M, Schurtenberger P (2016). Superresolution microscopy of the volume phase transition of pnipam microgels. Colloids and Surfaces A: Physicochem. Eng. Aspects.

[28] Wedel B, Zeiser M, Hellweg T (2012). Non NIPAM based smart microgels: Systematic variation of the volume phase transition temperature by copolymerization. Zeitschrift f. Phys. Chem..

[29] Wedel, B., Hertle, Y., Wrede, O., Bookhold, J. & Hellweg, T. Smart homopolymer microgels: Influence of the monomer structure on the particle properties. *Polymers* **8** (2016). 10.3390/polym8040162 PMC6432239 30979256

[30] Gan D, Lyon LA (2001). Tunable swelling kinetics in core-shell hydrogel nanoparticles. J. Am. Chem. Soc..

[31] Sleeboom JJF (2017). Compression and reswelling of microgel particles after an osmotic shock. Phys. Rev. Lett..

[32] Bertrand T, Peixinho J, Mukhopadhyay S, MacMinn CW (2016). Dynamics of swelling and drying in a spherical gel. Phys. Rev. Applied.

[33] Tanaka T, Fillmore DJ (1979). Kinetics of swelling of gels. J. Chem. Phys..

[34] Patil N, Soni J, Ghosh N, De P (2012). Swelling-induced optical anisotropy of thermoresponsive hydrogels based on poly(2-(2-methoxyethoxy)ethyl methacrylate): Deswelling kinetics probed by quantitative mueller matrix polarimetry. The Journal of Physical Chemistry B.

[35] Wang J, Gan D, Lyon LA, El-Sayed M (2001). Temperature-jump investigations of the kinetics of hydrogel nanoparticle volume phase transitions. J. Am. Chem. Soc..

[36] Keidel, R. *et al*. Time-resolved structural evolution during the collapse of responsive hydrogels: The microgel-to-particle transition. *Science Advances* **4** (2018). 10.1126/sciadv.aao7086 PMC5938240 29740608

[37] Grobelny S (2013). Conformational changes upon high pressure induced hydration of poly(*N*-isopropylacrylamide) microgels. Soft Matter.

[38] Reinhardt M (2013). Fine-Tuning the Structure of Stimuli-Responsive Polymer Films by Hydrostatic Pressure and Temperature. Macromolecules.

[39] Pelton RH, Chibante P (1986). Preparation of aqueous latices with n-isopropylacrylamide. Colloids and Surfaces.

[40] Berne, B. J. & Pecora, R. *Dynamic Light Scattering* (John Wiley & sons, Inc., New York, 1976).

[41] Stieger M, Pedersen JS, Lindner P, Richtering W (2004). Are thermoresponsive microgels model systems for concentrated colloidal suspensions? a rheology and small-angle neutron scattering study. Langmuir.

[42] Beyer A (2015). Imaging of carbon nanomembranes with helium ion microscopy. Beilstein Journal of Nanotechnology.

[43] Cors M (2017). Core-“shell microgel-based surface coatings with linear thermoresponse. Langmuir.

[44] Stieger M, Richtering W, Pedersen JS, Lindner P (2004). Small-angle neutron scattering study of structural changes in temperature sensitive microgel colloid. J. Chem. Phys..

[45] Kuttich B, Grillo I, Schöttner S, Gallei M, Stühn B (2017). Polymer conformation in nanoscopic soft confinement. Soft Matter.

[46] Müller A (2014). Kinetics of pressure induced structural changes in super- or near-critical Co_2_-microemulsions. Phys. Chem. Chem. Phys..

[47] Hellweg T, Kratz K, Pouget S, Eimer W (2002). Internal dynamics in colloidal pNIPAM microgel particles immobilised in a mesoscopic crystal. Colloids and Surfaces A.

[48] Wu C, Zhou S (1997). Volume phase transition of swollen gels: Discontinuous or continuous. Macromolecules.

[49] Nikolov S, Fernandez-Nieves A, Alexeev A (2018). Mesoscale modeling of microgel mechanics and kinetics through the swelling transition. Applied Mathematics and Mechanics.

[50] Hirano T (2008). Hydrogen-bond-assisted syndiotactic-specific radical polymerizations of *N*-alkylacrylamides: The effect of the *N*-substituents on the stereospecificities and unusual large hysteresis in the phase-transition behavior of aqueous solution of syndiotactic poly(*N*-*n*-propylacrylamide). Journal of Polymer Science Part A: Polymer Chemistry.

[51] Schürmann M (2015). Helium ion microscopy visualizes lipid nanodomains in mammalian cells. Small.

[52] Schindelin J (2012). Fiji: an open-source platform for biological-image analysis. Nature methods.

[53] Provencher SW (1982). A constrained regularization method for inverting data represented by linear algebraic or integral equations. Computer Physics Com..

[54] Provencher SW (1982). Contin: a general purpose constrained regularization program for inverting noisy linear algebraic and integral equations. Computer Physics Com..

[55] Koppel DE (1972). Analysis of macromolecular polydispersity in intensity correlation spectroscopy: The method of cumulants. J. Chem. Phys..

[56] Hassan P, Kulshreshtha S (2006). Modification to the cumulant analysis of polydispersity in quasielastic light scattering data. Journal of Colloid and Interface Science.

[57] Müller, A. *Preparation of polymer nano-foams: templates, challenges and kinetics* (Cuvillier Verlag, Göttingen, 2013).

[58] Pütz, Y. *CO*_2_*-microemulsions with additives: Phase behaviour, microstructure and pressure-induced kinetics* (Cuvillier Verlag, Göttingen, 2015).

